# Law Regulating the Practice of Dentistry in Alabama

**Published:** 1845-03

**Authors:** 


*Law Regulating the Practice of Dental Surgery in Alabama.*—We are indebted to Dr. Bellangee, of Montgomery, Alabama, for a copy of the law regulating the practice of dental surgery in that state. Having expressed our views in a preceding number of the Journal concerning the provisions of this law, it is unnecessary to say more on the subject. The following is a copy of the law.—*Balt. Ed.*

*An Act Regulating the Practice of Dental Surgery, and for other purposes.*

SECTION 1. *Be it enacted by the Senate and House of Representatives of the State of Alabama, in General Assembly convened,* That from and after the first Monday in December next, it shall be the duty of each of the medical boards of this state, to examine and license applicants to practice dental surgery, under the same rules and regulations, and subject to the same restrictions as those who apply for license to practice medicine; and, in order more fully to carry this act into effect, it shall be the duty of each of the medical boards, where the same is practicable, to add to their body, by election, a professional dentist, having the requisite qualifications, which dentist, so added, shall constitute a part of the board.

SEC. 2. *And be it further enacted,* That if any person, styling himself as dentist, or other person, shall engage in the practice of dental surgery as a professional business, after the aforesaid first Monday in December next, without having been regularly licensed so to do by one of the medical boards of this state, as hereinbefore provided for, for every such offence, shall forfeit and pay a sum not exceeding fifty dollars, recoverable before any court having jurisdiction of the same; one-half to the informer, the other half to the county where suit is brought.

SEC. 3. *And be it further enacted,* That all bonds, notes, or promissory obligations, or assumpsits, made to any person or persons not authorised as provided for in this act, the consideration of which shall be for services rendered as a professional dentist, or in the line of professional dentistry, shall be utterly void and of no effect: provided, the provisions of this act shall not be so construed as to prevent persons from practising dental surgery who have a license to practice surgery and medicine, from either of the medical boards of this state, or diploma from any regularly constituted medical institution in the United States.

SEC. 4. *And be it further enacted,* That hereafter it shall be the duty of all practising physicians, surgeons and dentists, to have their licenses recorded in the office of the clerk of the county court in which they may reside, and the certificate of the clerk shall be considered as good evidence in any court of the right of any individual, having a diploma or license to practice his profession, and recover his debts for the same.

SEC. 5. *And be it further enacted,* That all laws and parts of laws, contravening the provisions of this act, be and the same are hereby repealed.

APPROVED, December 31, 1841.

